# Editorial: Autophagy and Mitophagy in Skeletal Muscle Health and Disease

**DOI:** 10.3389/fphys.2021.703458

**Published:** 2021-06-09

**Authors:** Sabah N. A. Hussain, Marco Sandri, Gilles Gouspillou

**Affiliations:** ^1^Translational Research in Respiratory Diseases Program, Research Institute of the McGill University Health Centre (MUHC), Montreal, QC, Canada; ^2^Meakins Christie Laboratories, Department of Medicine, McGill University, Montreal, QC, Canada; ^3^Department of Biomedical Science, Veneto Institute of Molecular Medicine, University of Padova, Padua, Italy; ^4^Département des Sciences de l'activité Physique, Faculté des Sciences, Université du Québec à Montréal (UQAM), Montreal, QC, Canada; ^5^Centre de Recherche de l'Institut Universitaire de Gériatrie de Montréal (IUGM), Montreal, QC, Canada

**Keywords:** skeletal muscles, autophagy, proteolysis, atrophy, mitophagy, sympathethic nervous system

This Research Topic is dedicated to explorations of the functional roles of autophagy and mitophagy in skeletal muscles at molecular, cellular, and physiological levels. Autophagy is the cell's own waste management plant, where unneeded or dysfunctional components of the cell are disposed of or recycled. It is an orderly system, but extremely complex. There are several specialized structures and a wide array of regulatory molecules involved in autophagy. It plays a number of different functional roles depending on whether it is doing general housekeeping, assisting in host defense, reacting to cellular stress, taking part in embryonic development, or controlling the existential task of managing cell survival and cell death. The collection of papers we have assembled therefore covers a wide range of topics, including an examination of lysosomal enzyme activity in skeletal muscle cells, an inquiry into the effects of exercise on autophagic signaling pathways, a comparison of how mitophagy is regulated in non-muscle cell lines vs. skeletal muscles, an investigation into how sympathetic innervation regulates autophagy, and an overview of how autophagy maintains muscle cell homeostasis under physiological and pathological conditions.

## Autophagy in Skeletal Muscles

Skeletal muscle mass depends on a balance between protein synthesis and protein degradation. When muscle tissues are in a catabolic state, enhanced protein degradation leads to loss of muscle mass, a condition known as atrophy (Bonaldo and Sandri, 2013). Four proteolytic pathways govern this degradation process: the calpain, caspase, ubiquitin-proteasome, and autophagy-lysosomal pathways. In this Research Topic, we focus on the last one, the autophagy system, where cytosolic components and organelles are degraded by lysosomes. While four forms of autophagy have thus far been identified, for the present purposes, the term autophagy is synonymous with macroautophagy, which is the predominant form and the one that has been most thoroughly described in skeletal muscle physiology.

Autophagy is initiated by the ULK1 complex and a BECLIN1-dependent phagophore nucleation process follows, resulting in activation of local PI3P production. PI3P recruits the ATG protein WIPI2 and the ER-localized protein DFCP1 to the omegasome (a subdomain of the endoplasmic reticulum). WIPI2 binds directly to ATF16L1, triggering the ATG12-ATG5-ATF16L1 complex to promote the conjugation of ATG8 proteins, including LC3 and GABARAP, to membrane-resident phosphatidylethanolamine. Resulting membrane-bound lipidated forms of these proteins are a unique signature of autophagic membranes (Tanida et al., 2008). Bound molecules are transferred to the membrane, the membrane grows, and it eventually becomes a double-membraned vesicle known as an autophagosome (Klionsky, 2005). Fragments of damaged cytoplasm, organelles, protein aggregates, and other unneeded cellular components that have been sequestered by an autophagosome are then docked to a lysosome for degradation.

The functional importance of autophagy to skeletal muscle function and homeostasis is highly dependent on its degree and the duration of its inhibition. Chronic inhibition of basal autophagy leads to the development of fiber atrophy, decreased muscle force-generating capacity, accumulation of damaged organelles and proteins, and, ultimately, premature death (Masiero et al., 2009; Carnio et al., 2014). Acute inhibition of autophagy results in preservation of muscle mass. This is what occurs during acute starvation or in the case of severe oxidative stress (Mammucari et al., 2007; Dobrowolny et al., 2008).

## Contributions to the Research Topic

In an elegant original research project, Yun et al. evaluated the role of mitochondrial reactive oxygen species (mtROS) in the regulation of palmitoyl protein thioesterase 1 (PPT1). PPT1 is an enzyme responsible for the removal of palmitate from lysosomal proteins. It is essential to the mTORC1-TFEB signaling pathway and a specifically required component of the myogenic autophagy that occurs during muscle cell differentiation and regeneration. They demonstrated that PPT1 expression increases and its promoter is transactivated by Nrf2 during differentiation. They also show that PPT1 expression and activity is required for muscle cell differentiation and that PPT1 knockdown inhibits autophagy and attenuates the activities of cathepsin B, D, and K, three proteases that are essential to protein degradation within lysosomes. These findings strongly support the view that PPT1 plays an important regulatory role in autophagy and lysosomal function during the differentiation of myoblasts to myotubes.

Sarcopenia is the progressive loss of muscle mass and function that occurs during the aging process. It exerts considerable negative impacts on quality of life, physical ability, and life expectancy (Larsson et al., 2019). It is generally accepted that exercise mitigates damage, however, the relative efficacies of different forms of exercise and the mechanisms through they elicit positive results remain unclear. Zeng et al. evaluated the effects of four forms of exercise training on sarcopenia of limb muscles in aged rats. They report that exercise training in general, and resistance training in particular, suppress atrophy, increase autophagy-related protein expressions, and augment anti-apoptotic protein expressions. They also found that exercise training improves mitochondrial biogenesis and quality. These effects are attributed to inhibition of the AKT and mTOR pathways and activation of FoxO3 and the AMPK pathway, a crucial cellular energy sensor. Their study provides important clues as to the forms of training that would be most beneficial to elderly subjects suffering from sarcopenia and neatly delineates the mechanisms through which training works to improve muscle mass, quality, and function.

Mitophagy is the selective autophagy of mitochondria and is essential to the clearance of defective mitochondria since impaired mitochondrial function and integrity is a major factor in the development of muscle fiber atrophy in several pathologies, including sarcopenia (age-related loss of muscle mass), cancer cachexia, severe sepsis-associated muscle dysfunction (Drake and Yan, 2017; Leduc-Gaudet et al., 2020; Hyatt and Powers, 2021). In non-muscle cells, the PTEN-induced kinase 1 (PINK1)-Parkin pathway has been recognized as a major regulator of mitophagy. It is activated by a loss of mitochondrial membrane potential, raised levels of reactive oxygen species (ROS), and increased mitochondrial DNA damage (Narendra et al., 2008, 2010), leading to phosphorylation and activation of Parkin, an E3 ubiquitin ligase. Parkin ubiquitylates damaged mitochondria and tags them for recycling by autophagosomes. Seabright and Lai have integrated the most recent literature on PINK1-Parkin-mediated mitophagy in non-muscle cell lines to highlight mechanistic differences that seem to be unique to skeletal muscles. Their clear and concise formulation of a new model of mitophagy regulation in these muscles posits a prominent role for 5'-AMP-activated protein kinase (AMPK) and poses the question of whether or not mitochondrial ubiquitylation is even required for mitophagy in muscle. They suggest that one way of settling the issue would be to use *mito*-QC to quantify lysosome dependent mitochondrial degradation in conjunction with monitoring the signaling pathways that mediate mitochondrial fission and autophagosomal engulfment. We look forward to future key discoveries in mitophagy that they and others will make by using innovative biotechnologies like *mito*-QC.

Recent evidence indicates that neuromuscular junctions (NMJ) are co-innervated by sympathetic neurons (Khan et al., 2016), but studies on the role that this plays in skeletal muscle physiology are scarce. With the aim of furthering our understanding of how this affects autophagy, Straka et al. used a skillful combination of *in vivo* imaging, proteomics, and biochemical, and microscopic approaches to compare normal and sympathectomized hindlimb muscles of mice. They report that sympathectomized muscles display myofiber atrophy, increased acetylcholine receptor turnover, and increased content of endo/lysosomal acetylcholine-receptor-bearing vesicles. They also report that while sympathectomy led to a severe downregulation of mitochondrial proteins, it also enhanced markers of endocytic trafficking and autophagy, indicating that sympathetic innervation of NMJ exerts a regulatory role on autophagy in skeletal muscle cells.

In a comprehensive review article that provides an up-to-date overview of the molecular machinery involved in autophagy, Xia et al. summarize the key signaling pathways that regulate autophagy in skeletal muscles and outline various types of cross-talk between autophagy, protein synthesis, and the ubiquitin-proteosome pathway. They also survey the available literature on the pathological roles that impaired autophagy plays in muscle disorders, including various muscular dystrophies, Bethlem myopathy, Danon disease, X-linked myopathy with excessive autophagy, Pompe disease, and sporadic inclusion body myositis. Finally, they provide recommendations for future studies that emphasize the use of multiomic techniques to further our understanding of the underlying autophagy-related molecular and biochemical mechanisms of disease in skeletal muscle disorders.

In summary, the papers published in this Research Topic introduce readers to novel insights and important perspectives on the roles and regulation of autophagy and mitophagy in skeletal muscle health and disease. We sincerely hope that they will serve as a platform to stimulate new ideas and innovative research in the field.

## Author Contributions

All authors listed have made a substantial, direct and intellectual contribution to the work, and approved it for publication.

## Conflict of Interest

The authors declare that the research was conducted in the absence of any commercial or financial relationships that could be construed as a potential conflict of interest.
